# The Influence of Nanosilica on Properties of Cement Based on Tetracalcium Phosphate/Monetite Mixture with Addition of Magnesium Pyrophoshate

**DOI:** 10.3390/ma15228212

**Published:** 2022-11-18

**Authors:** Radoslava Stulajterova, Maria Giretova, Lubomir Medvecky, Tibor Sopcak, Lenka Luptakova, Vladimir Girman

**Affiliations:** 1 Division of Functional and Hybrid Systems, Institute of Materials Research of SAS, Watsonova 47, 040 01 Kosice, Slovakia; 2 Department of Biology and Physiology, University of Veterinary Medicine and Pharmacy in Kosice, Komenskeho 73, 041 81 Kosice, Slovakia

**Keywords:** hydroxyapatite composite, nanosilica, magnesium pyrophosphate, setting process, mesenchymal stem cells

## Abstract

The effect of nanosilica on the microstructure setting process of tetracalcium phosphate/nanomonetite calcium phosphate cement mixture (CPC) with the addition of 5 wt% of magnesium pyrophosphate (assigned as CT5MP) and osteogenic differentiation of mesenchymal stem cells cultured in cement extracts were studied. A more compact microstructure was observed in CT5MP cement with 0.5 wt% addition of nanosilica (CT5MP1Si) due to the synergistic effect of Mg_2_P_2_O_7_ particles, which strengthened the cement matrix and nanosilica, which supported gradual growth and recrystallization of HAP particles to form compact agglomerates. The addition of 0.5 wt% of nanosilica to CT5MP cement caused an increase in CS from 18 to 24 MPa while the setting time increased almost twofold. It was verified that adding nanosilica to CPC cement, even in a low amount (0.5 and 1 wt% of nanosilica), positively affected the injectability of cement pastes and differentiation of cells with upregulation of osteogenic markers in cells cultured in cement extracts. Results revealed appropriate properties of these types of cement for filling bone defects.

## 1. Introduction

Calcium phosphate/monetite powder mixture is a frequently used calcium phosphate cement due to its appropriate mechanical properties, controllable setting process, and advanced biological characteristics with calcium-deficient nanohydroxyapatite as the final product of transformation [1]. In addition, monetite has enhanced osteoconductive and biodegradable properties compared with hydroxyapatite (HAP) or brushite [2]. Mg is the most abundant minor element in bone formation (~6 mol% substitution) during the early stages of osteogenesis, and its content in bones decreases as the bone matures [3]. Magnesium deficiency adversely affects all metabolic stages of skeleton formation, inhibits bone growth, reduces the activity of osteoblastic and osteoclastic cells, and promotes bone fragility [4]. Moreover, magnesium deficiency influences osteoporosis because it promotes the production of hydroxyapatite, as well as the secretion and activity of parathyroid hormone [5]. On the other hand, an insignificant role of magnesium on mesenchymal stem cells (MSC) differentiation to the osteogenic line was found in Mg-Ca alloy extracts with a higher concentration of ions than in pure Mg alloy, but the synergistic effect of both elements on cell activity was showed [6]. Magnesium also indirectly affects bone mineralization through the activation of alkaline phosphatase (ALP) [7]. The magnesium calcium phosphate cement (MCPC) with optimal composition can be characterized by more complex properties due to the composite character of cement, such as high strength arising from magnesium phosphate, as well as the calcium phosphate component, which causes the suppression of cytotoxicity and improves the cement matrix properties. [8].

The addition of bioglass to calcium sulfate-based cement improves the mechanical properties and osteogenic potential of the composite [9]. Furthermore, the presence of silica in biomaterials has generally been shown to improve osteoblast adhesion and proliferation [10]. The silicon is in matrix formation in bone and cartilage, but silicon deficiency insignificantly influences extracellular matrix formation compared with the mineral component itself [11]. It has been shown that silicate ions released from silica-substituted calcium phosphates could positively affect osteoblasts [12,13,14,15,16,17,18,19]. Previous studies mostly focused on the use of high silica content from 25 wt% up to 73 wt% for the apparent effect of silica in CPC [20]. It was verified that a high silica content (up to 30% by weight) in the SiO_2_/βTCP composite powder increased ALP activity and OC production by human dental pulp cells, as well as an increase in the antibacterial activity of cements containing silica [21] due to the formation of Si–OH groups in SBF via SiO_2_ hydrolysis [15,22,23,24]. These Si–OH groups represent hydroxyapatite nucleation sites during bone healing. In addition, the release of SiO_4_ from nanosilica can also intensify the nucleation and precipitation of calcium phosphate in SBF.

The preparation of calcium phosphate cement (CPC) in injectable form can simplify the treatment of bone defects. In the case of injectable cements-methacrylate composites (disadvantages: the presence of free monomer, low bioactivity, non-absorbable, high temperature during polymerization), sulfate cements (disadvantages: quickly absorbable and with increased solubility in body fluids) and calcium phosphate cements (pH problems, longer setting time, problematic viscoelastic properties) are used in practice [25].

In this paper, the objectives were focused on the analysis of the influence of nanosilica (mixed in low contents into the basic calcium phosphate cement mixture in the form of a suspension with the liquid cement component (2% NaH_2_PO_4_ solution)) on the microstructure, the process of cement setting and the osteogenic differentiation of mesenchymal stem cells cultured in cement extracts and evaluate the current influence of a very low concentration of silicon and magnesium ions released from cement during setting to the solution on bioactivity. Note that in most works focused on the role of silicon on the osteogenic differentiation of MSCs, a relatively high concentration of silicon ions was used, but the low content of Si in bones indicates that even minute amounts of this element are biologically active during the early stages of bone growth [26,27,28].

## 2. Materials and Methods

### 2.1. Preparation of Cement Mixture and Samples for Evaluation

The equimolar tetracalcium phosphate (TTCP) and monetite (DCPA, calcium hydrogen phosphate anhydrous, Ph. Eur, Merck, Darmstadt, Germany) CPC powder mixture was prepared according to a previous method in Ref. [29]. 

Magnesium pyrophosphate was synthesized by decomposition of newberyite (MgHPO_4_·3H_2_O, Sigma Aldrich, Steinheim, Germany) at 1000 °C for 1 h. The CT5MP powder mixture was prepared by simple homogenization of CPC and Mg_2_P_2_O_7_ in a planetary ball mill (RETSCH GmbH, Haan, Germany, 200 rpm for 1 h, ethanol). The 1% and 2% colloidal nanosilica (99.8%, fumed silica, Sigma-Aldrich, St. Louis, MO, USA) suspension in 2% NaH_2_PO_4_ (analytical grade, Sigma-Aldrich, Steinheim, Germany) solution was used as a liquid component. The powder-to-liquid (P/L) ratio was 2. The cement pastes were packed in a stainless steel cylinder (6 mm D × 12 mm H) and hardened at 100% humidity at 37 °C for 10 min. Then the samples were soaked in simulated body fluid (SBF) at 37 °C for 1 week. The hardened cements were designated as CT (no nanosilica), CT1Si, and CT2Si (the liquid component contained 1 and 2 wt%, respectively), and so on CT5MP, CT5MP1Si, CT5MP2Si.

### 2.2. Characterization Methods

The compressive strength (CS) was measured on a universal testing machine (LR5K Plus, Lloyd Instruments Ltd., West Sussex, UK) at a crosshead speed of 1 mm/min (mean + standard deviation, *n* = 5). The phase composition of the samples was analyzed by X-ray diffraction analysis (Philips X PertPro, Malvern Panalytical B.V., Eindhoven, The Netherlands) using Cu Ka radiation, 50 mA, 2 Θ range 20–40°) and FTIR spectroscopy (Shimadzu, Kyoto, Japan, IRAffinity1). The microstructures of the cements were observed by field emission scanning electron microscopy (JEOL FE SEM JSM7000F, Tokyo, Japan) after carbon coating. The morphology of the particles in the samples was observed using transmission electron microscopy (JEOL JEM 2100F, Tokyo, Japan).

The final setting times (ST) of the cement pastes were evaluated using the tip (1 mm diameter) of a Vicat needle with a 400 g load (according to ISO standard 1566). The amount of released calcium, phosphate, magnesium, and silicate ions from cements during soaking of cement pellets in SBF solution at 37 °C (400 mg/15 mL solution, polypropylene tube and shaken) were determined by ICP OES (Horiba ActivaHoriba Activa, HORIBA Jobin Yvon Inc., Park Avenue, Edison, NJ, USA) after filtration over the membrane filter (PVDF, 45 µm pore size, Millipore) at selected soaking times (1, 2, 6, 24, 48, and 168 h).

The injectability of the cements was tested by the extrusion of cement paste from a polypropylene syringe. Briefly, a 10 mL syringe with a nozzle inner diameter of 2 mm was filled with cement pastes (approximately 5 mL) after mixing the cement powder mixture with hardening liquid at a given P/L ratio. The paste was manually extruded from the syringe 3 min after the addition of the liquid component. The cement injectability represents the relative mass amount of extruded paste to the origin mass of cement paste in a syringe. The injectability was expressed as mean ± SD (*n* = 4).

### 2.3. In Vitro Cytotoxicity Testing of Mg-Si Cement Extracts

For the determination of cytotoxicity, the cement extracts were prepared as follows. Cement pastes were soaked in a complete osteogenic differentiation culture medium (α-modification minimum essential medium Eagle, α MEM; Biosera, Hoddesdon, UK), 10% FBS (Biowest, Niayers, France), 50 μg/mL of L-ascorbic acid, 50 nM dexamethasone, 10 mM β-glycerophosphate and 1% penicillin, streptomycin, and amphotericin (all Sigma-Aldrich) in an incubator at 37 °C for 24 h ((0.2 g cement paste/mL of culture medium) (ISO 10993-12:2012 [30] 100% extracts). The extracts were used for 24 h in vitro cytotoxicity testing using pre-osteoblastic cell line MC3T3E1 Subclone 4 cells (ATCC CRL-2593, Manassas, VA, USA). The density of cultured MC3T3E1 cells was adjusted to 1.0 × 10^5^ cells/mL. The 1.0 × 10^4^ cells in 100 μL of medium (α MEM + 10% FBS, 1% antibiotic solution) were seeded to wells of a 96-well microplate (cell grade Brand, adherent wells), and cultured in an incubator for 24 h. Then the culture medium was replaced with 100 μL of prepared extracts, and cultivation took the next 24 h in an incubator at 37 °C, 95% humidity, and 5% CO_2_. Finally, cytotoxicity was examined using the MTS proliferation test (Cell Titer Aqueous One Solution Cell Proliferation Assay, Promega, Madison, WI, USA) according to ISO 10993–5:2009 [31]. The formazan produced by viable cells was quantified by measuring the absorbance at 490 nm on a UV–VIS spectrophotometer (Shimadzu UV 1800, Kyoto, Japan). Sterilized cement samples (Ø 6 mm, 1 mm thickness) were placed in 48–well culture plate wells. To each tested sample were added 2 × 104 MC3T3E1 cells in 400 μL of culture medium. After 2 days of culturing, the density, distribution, and morphology of the cells were assessed by live/dead fluorescent staining (fluorescein diacetate FDA/propidium iodide PI) by an inverted optical fluorescence microscope (Leica DM IL LED, Heerbrugg, Switzerland blue filter).

For long-term cytotoxicity evaluation, ALP activity, and gene expression, the rat MSCs isolated from the bone marrow of rats’ femur and tibia were used and characterized [32]. 

For long-term cytotoxicity testing of cement extracts (up to 14 days), a ratio of 0.1 g cement paste/mL culture medium (50% extracts) was selected. The extraction medium also consisted of a complete osteogenic differentiation culture medium α-MEM + osteogenic supplements (mentioned above). The extraction was carried out in an incubator at 37 °C for 24 h. The cell density of resuspended rat MSCs passage 3 in culture medium was adjusted to 5.0 × 10^4^ cells/mL, and 2.0 × 10^4^ of rat MSCs in 400 μL of αMEM + 10% FBS, 1% antibiotic solution was seeded into each well of a 48-well plate (TPP, Trasadingen Switzerland). After cultivation for 24 h in an incubator to a semi-confluent monolayer of cells, the culture medium in the wells was replaced with 400 μL of prepared extracts (0.1 g/mL). All cytotoxicity tests were carried out in triplicate, and cells cultured in extract-free complete culture medium were considered the negative control. Extracts were renewed three times a week. 

The ALP activity of osteogenic differentiated rat MSCs cultured in long-term extracts was determined in cell lysates. The solutions of equal amounts of cell lysate and phosphatase substrate were incubated at 37 °C for 60 min. The ALP activity was expressed by the amount of p-nitrophenol produced during the ALP enzyme catalysis and was determined using the UV VIS spectrophotometer at 405 nm. The quantification of proteins in lysates was evaluated by Bradford’s assay. Alizarin red staining was used to visualize the production of calcium deposits by differentiated osteogenic cells. Cells in extracts were washed with PBS, fixed in ethanol, and washed twice with dH_2_O. Deposits were stained with Alizarin red S staining solution for 30 min. After removing the staining solution and washing, the cells were observed under a light microscope (Leica DM IL LED, Heerbrugg, Switzerland). 

### 2.4. Gene Expression of Specific Markers in Differentiated Rat MSCs in Long-Term Culture

The gene expression was analyzed according to the method in Reference [33]. For the extraction of total RNA, 1 × 10^6^ cells were used. The quantification of genes in the cDNA samples was performed using primers for the following genes: B-actin rat, type I collagen rat, osteocalcin rat, osteopontin rat, osteonectin rat, and alkaline phosphatase rat in Table 1 [34,35,36,37,38]. cDNA for β actin was used as the endogenous control for calculating fold differences in the RNA levels of cells treated vs. not treated with cement extracts according to the 2^−∆∆CT^ method. The plate was sealed using an optical adhesive cover (Roche, Basel, Switzerland) and placed in a LightCycler 480 II real-time PCR system machine (Roche, Basel, Switzerland). Real-time PCR was performed under the following conditions: initial incubation at 95 °C for 10 min, amplification in 45 cycles at 95 °C for 15 s, followed by 60 °C for 1 min. Amplification specificity was checked by the generation of a melting curve.

## 3. Results

### 3.1. XRD and FTIR Analyses of Powder Mixtures and Cements

**Figure 1:**
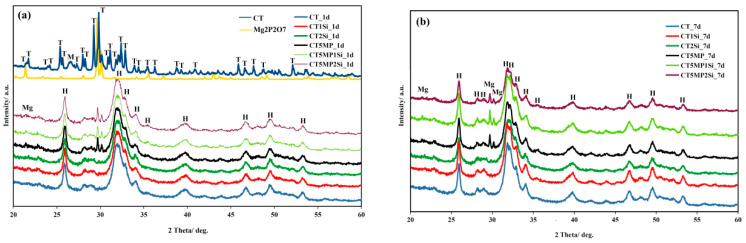
XRD patterns of origin CT5MP powder mixture and cements after 1 (**a**) and 7 days setting (**b**) in SBF at 37 °C. (Mg: Mg_2_P_2_O_7_ (JCPDS 32-0626), H: hydroxyapatite (PDF4 01-071-5048), M: monetite (JCPDS 09-0080), T: TTCP JCPDS 25-1137)).

The XRD patterns of the powdered CT and CT5MP mixture and hardened cements after one and seven days of soaking in SBF are shown in Figure 1. In the XRD patterns of the CT and CT5MP mixture, there were found lines originating from the reflection of TTCP (JCPDS 25-1137), monetite (JCPDS 09-0080, reflections from planes (020), (−220), and (−112) at 2Θ~26.51 and 30.21°) and magnesium pyrophosphate (JCPDS 32-0626, the most intense reflections from the (012) and (301) planes) starting phases were clearly identified. A comparison of the samples showed complete conversion of the calcium phosphate phases in both CT (without Mg_2_P_2_O_7_) and CT5MP cements to nanocrystalline hydroxyapatite (PDF4 01-071-5048) even after 24 h of soaking in SBF, in contrast to the magnesium pyrophosphate phase, which was only gradually hydrolyzed (about 15% of the amount of Mg_2_P_2_O_7_ in CT5MP was hydrolyzed between two and seven days of soaking) and converted to Mg substituted hydroxyapatite after soaking CT5MP cements for seven days in SBF. No dependence of the average crystallite size in hydroxyapatite particles with the addition of Mg_2_P_2_O_7_ or nanosilica in cements was found, but increasing the soaking time from one to seven days in SBF clearly promoted the recrystallization of HAP nanoparticles in cements from 24 nm to 33 nm (calculated from the (002) line of HAP using the Scherrer equation).

**Figure 2:**
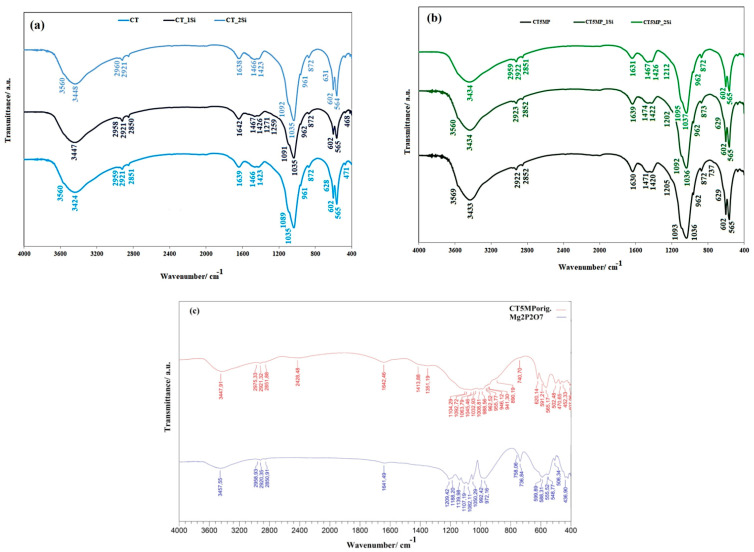
FTIR spectra of (**a**) CT powder mixture and CT1Si, CT2Si cements after 7 days soaking in SBF; (**b**) CT5MP powder mixture and CTMP1Si, CTMP2Si cements after 7 days soaking in SBF; (**c**) CT5MP origin powder mixture before soaking in SBF and Mg_2_P_2_O_7_.

The FTIR spectra verified the results from the analysis of XRD patterns of cements after seven days soaking in SBF, where full transformation of starting calcium phosphate phases to nanocrystalline hydroxyapatite (Figure 2) was identified. Novibrations from a symmetric stretching (962–941 cm^−1^) mode (ν_1_) of TTCP [39,40] or ν_3_ stretching vibrations of P–O and P–O(H) monetite bonds (at 1134 and 900 cm^−1^) as well as the O-H plane bending monetite vibrations (at 1410 and 1351 cm^−1^), were found [41]. Remains of the Mg_2_P_2_O_7_ phase characterized by the presence of very low intense peaks at 1210 and 735 cm^−1^ [42,43,44] were only visible in spectra. The shape of the FTIR spectra of cements indicates a low crystallinity of HAP nanoparticles due to almost unresolved bands arising from the stretching and librational vibrations of the OH group in hydroxyapatite at 3560 cm^−1^ and 630 cm^−1^, respectively. On the other hand, the ν_1_ (at 962 cm^−1^), ν_4_ (at 602 and 567 cm^−1^), ν_2_ (at 471 cm^−1^), and ν_3_ vibrations (at 1089 and 1037 cm^−1^) arise from vibrations of the phosphate group in hydroxyapatite [45]. The location of carbonate bands at ~1470, 1420, and 873 cm^−1^ (ν_3_ asymmetric stretching and ν_2_ out of plane bending vibrational modes) clearly shows the formation of B-type carbonated hydroxyapatite [45,46].

### 3.2. Microstructure of Cements and Morphology of HAP Particles

**Figure 3:**
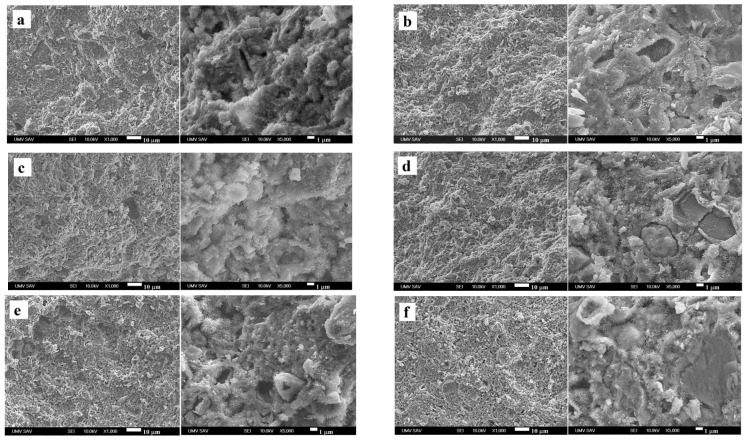
Microstructure of fractured cements after 7 days of hardening in SBF at 37 °C: CT (**a**), CT1Si (**b**), CT2Si (**c**), CT5MP (**d**), CT5MP1Si (**e**), CT5MP2Si (**f**).

**Figure 4:**
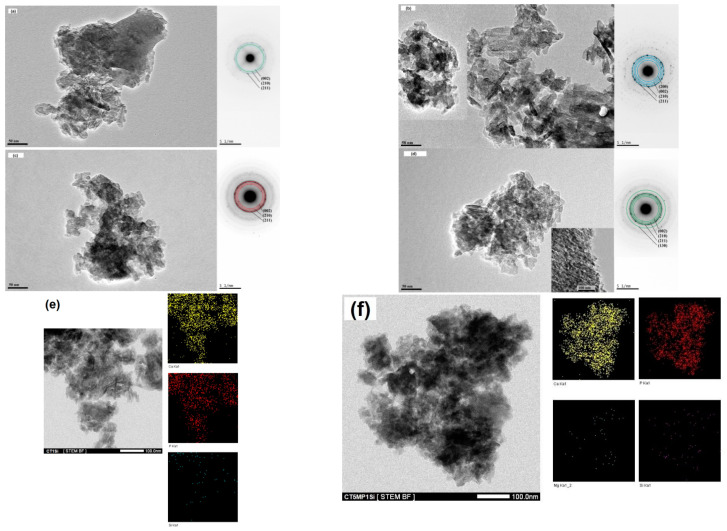
Morphology and SAED of hydroxyapatite nanoparticles in CT (**a**), CT1Si (**b**), CT5MP (**c**), and CT5MP1Si (**d**) observed using TEM and STEM/EDX analysis (mapping) of elements in CT1Si (**e**), CT5MP1Si (**f**).

The microstructure of fractured CPC cements was affected by the addition of nanosilica to the cement mixture, as can be seen in (Figure 3a–c). The fraction of micropores with a size >2 µm increased with the amount of nanosilicate in the cement, and the relatively dense microstructure of the original CT cement with needle-like thin HAP nanoparticles up to 50 nm in length (Figure 4a) changed to a microstructure formed by coarser agglomerates with two morphological types of nanoparticles–spherical and longer needle-like HAP nanoparticles (up to 70 nm) (Figure 4b) in the form of compact layers around the original TTCP particles transformed into HAP during soaking (Figure 3b). Higher contents of nanosilica clearly supported the formation of globular agglomerates of size 1–3 µm composed of HAP nanoparticles of mixed morphology (further thickening of needle-like HAP particles was observed in CT2Si cement) and weakly interconnected (Figure 3c). Please note that EDX analyses verified the homogeneous distribution of silicon in the cements, which is in accordance with the procedure used for the preparation of cement pastes.

In CT5MP cement, a shortening of fine needle-like HAP particles was identified in the cement matrix (Figure 4c), as well as a higher fraction of spherical nanoparticles (20–30 nm in size) compared with CT cement. In addition, larger dense HAP agglomerates of 5–10 µm (no increased magnesium content was confirmed by EDX analysis) were surrounded by a layer of needle-like HAP nanoparticles, which were often separated from the agglomerates by a visible depletion zone (Figure 3d). A significant coarsening of needle-like particles was found with nanosilica content in CT5MP cement up to 100 and 200 nm in CT5MP1Si (Figure 3e) and CT5MP2Si (Figure 3f). On the other hand, the microstructure of CT5MP1Si was more compact, with no visible separation of agglomerates from the matrix and a tight connection of nanoparticles in the agglomerates (Figure 4d, detail) in contrast to the microstructure of CT5MP2Si, where a similar mutual separation of globular HAP agglomerates was identified as in the case of CT2Si cement.

**Figure 5:**
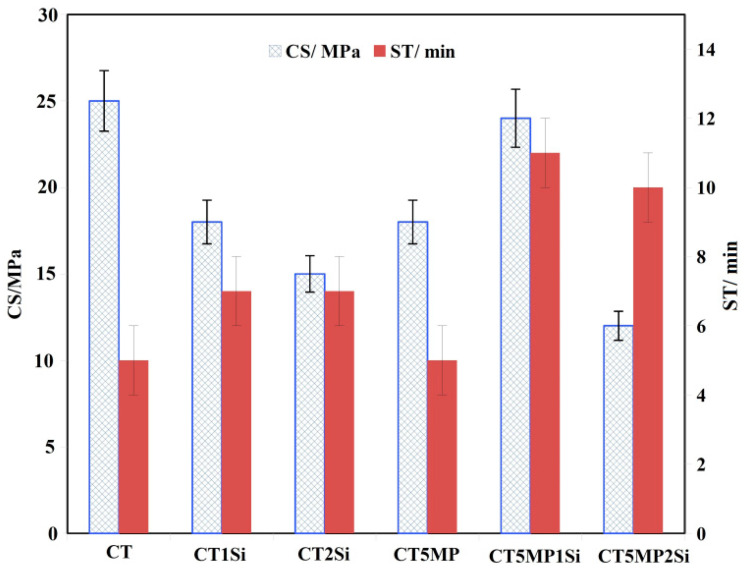
Setting time and compressive strength of cements after 7 days hardening in SBF at 37 °C.

The relative density of the cement was close to 46 ± 2% of the theoretical HAP density, and no statistically significant differences (*p* > 0.51) were measured between the mean relative density of the samples. In the case of CT, the ST increased from 5 to 7 min by adding nanosilica, but the simultaneous presence of magnesium pyrophosphate actively affected the ST increase in CT5MP up to 10–11 min (Figure 5). On the other hand, the CS (Figure 5) decreased with the concentration of nanosilica in cements from 25 to 15 MPa in CT and CT2Si cements. The addition of 0.5 wt% of nanosilica to CT5MP cement caused an increase in CS from 18 to 24 MPa (statistically significant difference, *p* < 0.03) for CT5MP and CT5MP1Si cements, while an almost two-fold reduction in CS (up to 12 MPa) was measured in CT5MP2Si containing 1 wt% nanosilica.

In terms of evaluating the injectability of cements, 100% injectability was measured for all cements with the addition of nanosilica or magnesium pyrophosphate, in contrast to approx. 76 ± 4% of CT cements. CT1Si and CT5MP1Si cement pastes were additionally resistant to disintegration in an aqueous environment.

### 3.3. Release of Ca^2+^, Mg^2+^, Silicate, and Phosphate Ions from Cements to SBF and pH Measurement

**Figure 6:**
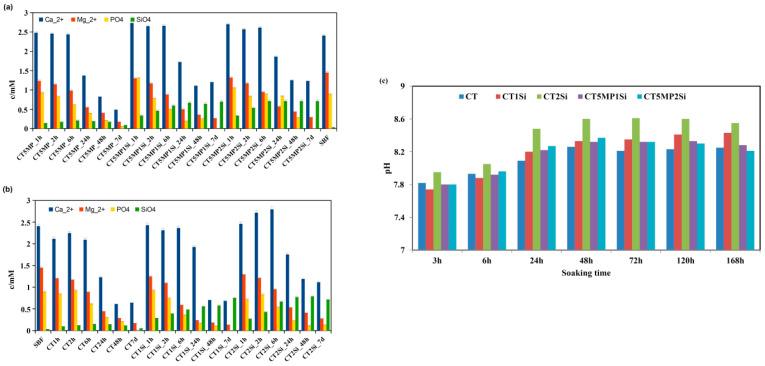
Release of Ca^2+^, Mg^2+^, silicate, phosphate ions (**a**) CT5MP, CT5MP1Si, CT5MP2Si, (**b**) CT, CT1Si, CT2Si and (**c**) change of pH during the soaking of cements in SBF at 37 °C.

The release of Ca^2+^, Mg^2+^, silicate, and phosphate ions from the cements during immersion in SBF at 37 °C is shown in Figure 6a,b. A small decrease in the concentration of Ca^2+^ ions in SBF with CT and CT1Si cements was measured only during the first 6 h of soaking, followed by a rapid decrease (almost half of the original concentration) and reaching a plateau equal to approximately 0.6 mM after 48 h of setting. A gradual decrease in the concentration of Mg^2+^ and phosphate ions with soaking time was identified in the solution with CT, CT1Si, and CT2Si cements. In the case of CT2Si, the concentration of calcium ions increased up to 6 h of soaking, and after 24 h, a rapid decrease with a concentration and plateau at about 1 mM Ca^2+^ was found for longer soaking times. In both solutions with cements containing nanosilica, the concentration of silicate ions increased steadily to approximately 0.6 mM after 24 h of soaking. Similar dependences of magnesium and silicate ions on soaking time were verified in CT5MP cements, but the concentration of Ca^2+^ ions during the first 6 h of soaking the cements with nanosilica was higher than the starting concentration in SBF and the plateau was close to 1.1 mM in CT5MP1Si and CT5MP2Si solutions in contrast from ~0.5 mM in CT5MP solution. In addition, the content of phosphate ions in CT5MP2Si solution was significantly higher than CT5MP and CT5MP1Si solutions up to 24 h of soaking. The pH of the solutions increased with soaking time and reached a maximum after 48 h close to 8.3 for CT, CT1Si, CT5MP1Si, and CT5MP2Si solutions. The pH of the CT2Si solution was statistically significantly higher than others (*p* < 0.02) for all selected soaking times (Figure 6c). In addition, a higher increase in pH with Si content was found in CT cement. This effect was not measured in CT5MP cements.

### 3.4. In Vitro Testing of Cement Extracts, Live/Dead Staining of Cells, Gene Expression

**Figure 7:**
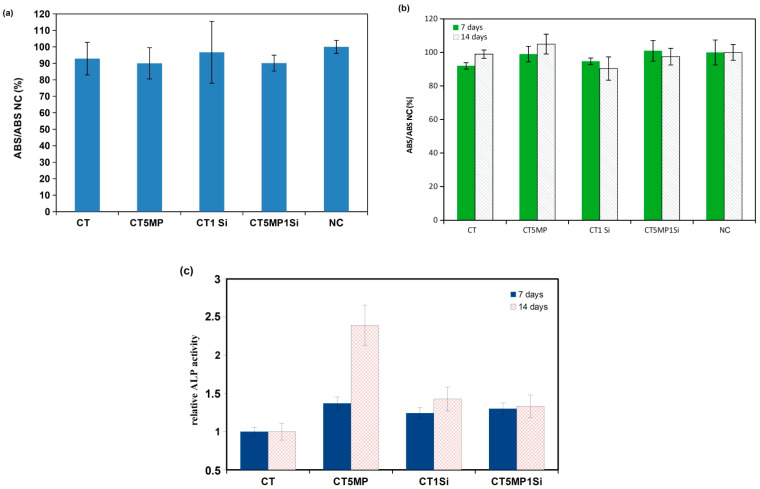
Viability of MC3T3E1 cells (**a**) cultured in 100% cement extracts for 24 h; viability of differentiated rat MSCs cultured (**b**) and ALP activity (**c**) of differentiated rat MSCs cultured in 50% cement extracts for 7 and 14 days.

In vitro cytotoxicity of 100% cement extracts was evaluated after 24 h of culture in an osteogenic medium (according to ISO 10993–5:2009 [31]) (Figure 7a). The relative cell viability of MSCs after 24 h of culture in all cement extracts reached >90% cell viability in NC (negative control), indicating their non-cytotoxic nature. A similar conclusion was demonstrated during the long-term culture of MSCs in 50% cement extracts (Figure 7b). A standard assay of ALP activity of MSCs differentiated in an osteogenic medium clearly identified the highest relative ALP activity in cells cultured in CT5MP extract compared with others, where the addition of nanosilica to the cements caused a decrease in ALP activity of the cells. In contrast to this fact, ALP activity culture in an extract of CT cements with the addition of nanosilica was higher than in cells cultured in CT extract (statistically significant difference, *p* > 0.05); thus, an increase in the content of silicate ions in the extract, even in a small amount, increased the ALP activity of cells by about 30–50% with the time of cultivation (Figure 7c).

**Figure 8:**
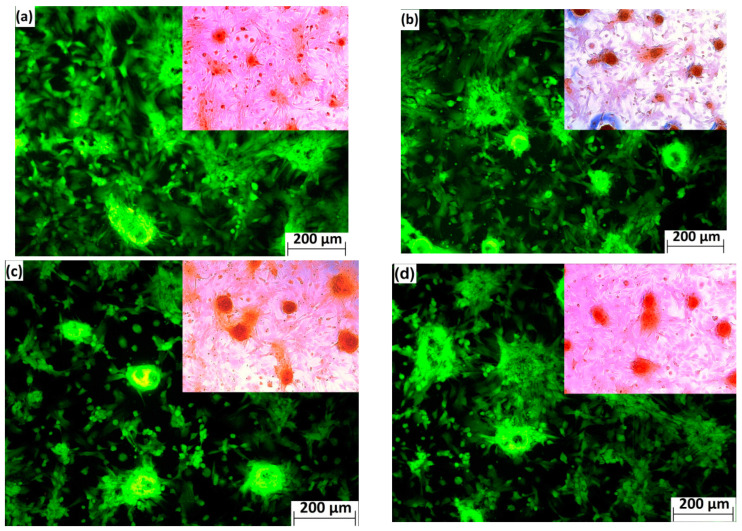
Morphology and distribution of MC3T3E1 osteoblasts cultured on CT (**a**), CT5MP (**b**), CT1Si (**c**), and CT5MP1Si (**d**) cement surfaces for 48 h (live/dead staining) and production of calcium deposits by differentiated rat MSCs cultured in 50% cement extracts for 14 days in details (Alizarin red staining).

A high number of viable cells was observed on the cement surfaces after two days of culture, and the cells were assembled into bundles interconnected by filopodia. Alizarin red staining verified the production of calcium deposits by differentiated rat MSC cells after fourteen days of culture in 50% cement extracts, indicating their osteogenic activity (Figure 8).

**Figure 9:**
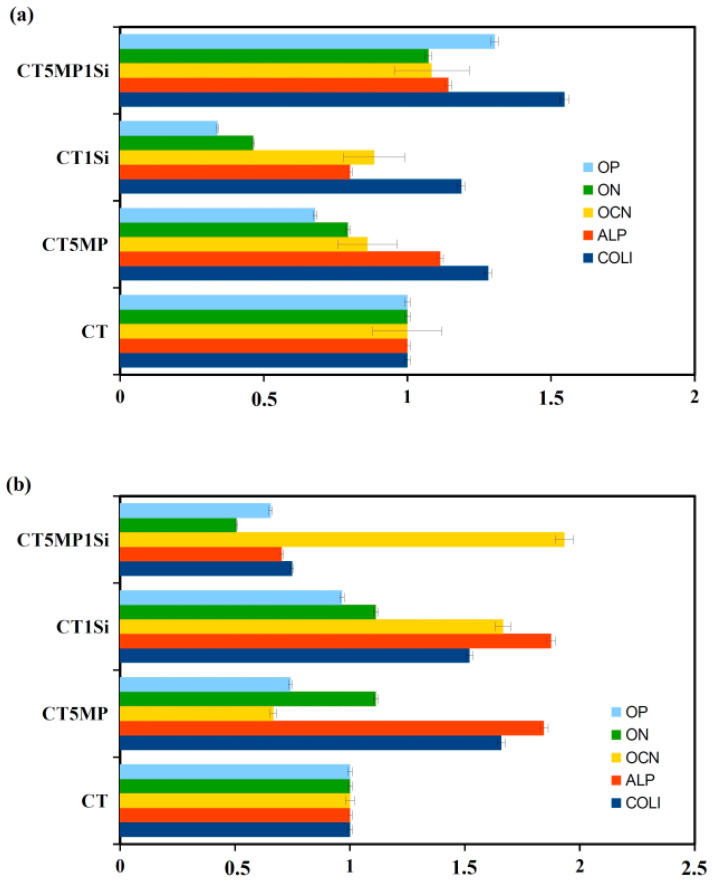
Relative gene expression of OP, ON, OCN, ALP, and COL1 in MSCs cultured for 7 (**a**) and 14 (**b**) days.

Gene expression RT PCR (Figure 9a) resulted in statistically significant up-regulation (*p* < 0.001) of ALP and COLI (collagen I) in cells cultured in CT5MP, CT1Si, and CT5MP1Si extracts for seven days compared with cells cultured in CT extract, but the highest overexpression of COLI gene was indicated in cells cultured in CT5MP1Si extract. After fourteen days of culture (Figure 9b), cells cultured in CT5MP and CT1Si cement extracts showed an increase in gene expression and upregulation (*p* < 0.0002) of COLI and osteogenic genes such as ALP, osteocalcin (OCN), and osteonectin (ON) whereas overexpression of OCN was only measured in cells after fourteen days of culture in CT5MP1Si.

## 4. Discussion

The addition of silica nanoparticles to CT and CT5MP was used to influence the setting process, mechanical properties, and microstructure. The results from XRD and FTIR analysis of hardened cements clearly showed the formation of carbonated nanocrystalline hydroxyapatite B-type after soaking in SBF solution with a relatively rapid and complete transformation of calcium phosphate phases after 24 h. This fact demonstrated small changes in the calcium concentration in SBF during 24 h of setting with a gradual consumption of phosphate ions and an increase in pH due to the faster hydrolysis of the TTCP phase, as well as the presence of a thin cement matrix depleted zone around the original TTCP particles. On the other hand, as shown in the previous article [47], hydrolysis and reactivity of magnesium pyrophosphate were much slower during soaking, which confirmed its residues in hardened cements after seven days of soaking. It was found that the transformation of components in the TTCP/brushite cement mixture was improved by the addition of 5 wt% of nanosilica, where almost pure nanohydroxyapatite was identified after seven days of soaking in SBF in contrast to the remains of the TTCP phase in cement without additives [15].

Note that in terms of typical microstructural features, CT cement was characterized by a more compact microstructure with a high proportion of thin needle-like nanohydroxyapatite particles as compared with CT1Si and CT2Si microstructures, where coarsening of needle-like HAP particles and the precipitation of spherical HAP nanoparticles connected to the form of weakly interconnected globular agglomerates were observed. Similarly, the elongation of needle-like particles of HAP-containing nanosilica was detected in CT5MP cements. Thus, the addition of silica nanoparticles to CT cement, even in a relatively small amount (1 wt%), promoted the growth of HAP particles and, in addition, the nucleation of a large number of fine spherical particles in CT5MP cements. This fact, also verified by EDX analysis, clearly indicates that the nanosilica was homogeneously distributed in the cement matrix. Probably the reason for the formation of the finer matrix was the presence of magnesium ions with an increased concentration of magnesium in the local environment of Mg_2_P_2_O_7_ particles and micropores in the cement matrix, which supported the refinement of HAP particles and, secondly, the specific properties of nanosilica with an acidic surface able to adsorb of metal ions like Ca^2+^ or Mg^2+^. 

In addition, a theoretical calculation focused on the analysis of the energy of formation and the favorable substitution of silicon in the hydroxyapatite lattice showed that orthosilicic acid can be incorporated into HAP as a silicate ion replacing a phosphate ion as well as probably under specific conditions at bone remodeling interface after metabolizing Si dietary forms into SiO_4_^4−^ anions [48].

Considering that magnesium consumption started in CT5MP from the first stages of setting regardless of the content of magnesium pyrophosphate in the cement, it is clear that the reason for the magnesium content decrease is the low solubility constant and the insufficient rate of hydrolysis of Mg_2_P_2_O_7_ in relation to achieving a stable concentration of Mg^2+^ in the solution. In contrast to the above facts, Mg was uniformly distributed in the hydroxyapatite lattice due to an almost linear decrease in its concentration during 24 h of hardening. It was shown that the dissolution of pyrophosphates in cements could be actively stimulated by an enzyme reaction with ALP, which promoted their transformation into orthophosphates affecting their biodegradation in vivo [49]. In addition, nanosilica can also be adsorbed onto TTCP particles with a strongly basic surface character to control the dissolution/precipitation processes in cements, especially the nucleation and growth or recrystallization of HAP particles during setting. Both processes-the adsorption of calcium ions by nanosilica, which is related to the slowing down of the diffusion rate of calcium ions from hydrolyzed TTCP over the nanosilica layer-can delay the onset of setting and prolong the setting time of cements, as was measured, due to insufficient saturation of the local microenvironment in wet cement with calcium ions in relation to the HAP solubility constant. These results are in contrast to the strong ST reduction found in αTCP and TTCP/brushite CPC with the addition of 2.5 wt% and 5 wt% of silica, but a different base or liquid component was used in these cements [15,50]. Moreover, a small increase in calcium concentration (statistically significant difference, *p* < 0.05) was measured in soaking solutions with nanosilica CT or CT5MP cements, which may possibly be related to the chemical equilibrium between the calcium/nanosilica/hydroxyapatite complex and calcium ions at a given pH. The stability of the calcium/nanosilica complex is lower, which corresponds to a higher concentration of calcium and silicate ions in the solutions for a longer soaking time and a more basic pH, where the stability of the complex decreases with the following desorption and dissociation of the complex simultaneously with an increase in the solubility of nanosilica particles.

Besides this, the presence of Mg in a medium with HAP powder enhances HAP solubility at given conditions with an increase in Ca^2+^ concentration [51]. Such conditions stimulate recrystallization with the growth of HAP particles as well as the formation of a larger amount of spherical nanoparticles in the initial setting due to a high number of active centers for HAP nucleation on nanosilica particles. Note that the solutions after 24 h of soaking were probably saturated with respect to silicate ions, as no differences were found between the silica concentration in cement solutions with different nanosilica content. The silicon-substituted TCP/HAP biphasic ceramics with up to 3 wt% Si substitution supported the precipitation of a new apatite phase on the surface in contact with physiological fluids faster than ceramics without silicon [52], which clearly indicates the effect of silica on the nucleation of the HAP phase. Besides, a 4 mM concentration of dissolved magnesium phosphate effectively reduced nucleation, but a higher concentration affected the growth of HAP particles, which is the opposite effect of silicon [53]. On the other hand, intensive precipitation of the HAP phase on the surface of magnesium silicate discs during immersion in SBF was verified with a relatively strong degradation of the samples and the corresponding release of magnesium or silicate ions [54]. Thus, a much higher concentration of both ions than in our solution should not have a negative effect on the precipitation of HAP particles.

In the case of CS, the formation of an enhanced number of globular HAP agglomerates weakly interconnected with the fine cement matrix in the nanosilica CT cements caused the reduction of CS cements, despite the presence of longer needle-like HAP nanoparticles connected in agglomerates. On the other hand, a more compact microstructure was observed in the case of CT5MP1Si cement due to the synergistic effect of Mg_2_P_2_O_7_ particles, which strengthen the cement matrix, as well as nano-silica, which promotes the gradual growth and recrystallization of HAP particles in the form of compact agglomerates. Moreover, the higher fraction of nano-silica particles in cements allowed to reduce the distances between individual nano-silica particles with possible partial polymerization through interconnected silicate groups, which may support the formation of a larger center for the precipitation of HAP particles and their aggregation into separated globular agglomerates. At neutral pH, the solubility limit for amorphous silica is around 100 ppm (1 mM), and condensation of silicate ions started at pH near 7 with mostly neutral Si(OH)_4_ species, followed by Ostwald ripening at higher pH [55,56]. Note that the condensation and agglomeration of silicate ions can increase the presence of metal ions. Similar polymerization was predicted in CPC with the addition of 5 or 10 wt% of nano-silica with compaction of the microstructure and increase of CS cements [15]. These facts contradict our results, which may be due to the lower content of nano-silica in the samples. In addition, Mg- or Si-substituted HAP particles were shown to have more acidic surfaces than pure HAP, which may improve the adsorption of calcium ions and the subsequent growth of HAP particles [57].

There was no cytotoxicity of CT cements regardless of the Si content in their extracts, as well as a positive effect of a low concentration of silicate ions (~0.6 mM) in extracts from cements containing nanosilicate on the ALP activity of MSCs during immersion, on the other hand, a significant decrease in the ALP of cells was found in CT5MP extracts. Silicate ions stimulated MSCs to osteogenic differentiation and the production of collagen I characteristic of immature osteoblasts during seven-day culture in cementum extracts, which demonstrated COLI gene regulation, while in MPC extracts, in addition to ALP, increased regulation of COLI genes (without nanosilica) was identified, which better osteogenic stimulation of cells was verified. A higher concentration of silicon in connective tissues and bones than in non-connective tissues was measured, as well as an enhanced concentration of Si in younger rats and demineralized bones, which showed a direct effect of Si on collagen turnover [58,59]. On the other hand, the overexpression of all measured MSC osteogenic genes was demonstrated in the CT5MP cement extract with the addition of nanosilica. After fourteen days of MSC culture, increased overexpression of COLI, ALP, ON, and OCN genes was found in CT1Si and CT5MP cementum extracts, but the OCN gene was upregulated only in CT5MP1Si extract. Thus, prolonged cell culture in CPC extracts with silicate ions successfully induced cells to osteoblastic differentiation and maturation, but CT5MP1Si extract allowed earlier cell maturation based on the results of RT PCR analysis. It has been shown that Si alone is not sufficient to initiate the mineralization process in cells, but soluble Si at a low concentration of 25 µg/mL (~0.9 mM) can stimulate factors and signals important for osteoblastic differentiation when combined with osteogenic medium [60]. On the other hand, the 4 mM concentration of Si was identified as optimal for the stimulation of proliferation and production of calcium deposits, whereas 6 mM concentration caused enhanced apoptosis of cells during cultivation [61]. The upregulation of the ON gene and high ALP activity of osteoblasts were revealed after the cultivation of composite SiO_2_/Ca_2_P_2_O_7_ ceramic discs [62]. Moreover, it was identified a correlation between Mg content (5–20 wt% MgO) in TTCP/DCPA cement mixture and cell adherence or gene expression where cells cultured on 5 wt% Mg cement mixture showed enhanced adsorption of fibronectin as well as overexpression of collagen I, OC, and ALP as osteogenic gene markers and due to an insignificant effect of enhanced extracellular concentration of Ca^2+^ and Mg^2+^ ions (~2.5 mM) on MSCs differentiation, suggesting that cellular activity probably resulted from direct contact of cells with insoluble Mg compounds (microcrystalline Mg_3_(PO_4_)_2_) in cements [63]. 

## 5. Conclusions

The addition of silica nanoparticles (0.5 resp. 1 wt%) to CT and CT5MP was used to influence the setting process, mechanical properties, microstructure, and bioactivity of mesenchymal stem cells. The results from XRD and FTIR analysis of hardened cements clearly showed the formation of carbonated nanocrystalline hydroxyapatite. The addition of 0.5 wt% of nanosilica to CT5MP cement caused an increase in compressive strength from 18 to 24 MPa. On the other hand, the compressive strength decreased from 25 to 15 MPa in CT and CT2Si cements with the concentration of nanosilica and without magnesium in cements. The injectability of cements was 100% for all cements with the addition of nanosilica or magnesium pyrophosphate, in contrast to approx. 76 ± 4% of CT cements. CT1Si and CT5MP1Si cement pastes were additionally resistant to disintegration in an aqueous environment.

From the above facts, it is evident that nanosilica addition to CT resp. CT5MP cement, even in a low amount, positively affected the injectability of cement pastes and osteogenic markers in cells cultured in cement extracts.

## Figures and Tables

**Table 1:** Forward (F) and reverse (R) primers of genes used for RT-PCR experiments.

Genes	Primers	References
B-actin rat	F: GTAGCCATCCAGGCTGTGTT R: CCCTCATAGATGGGCAGAGT	[34]
Typ I collagen rat	F: CCAGCTGACCTTCCTGCGCC R: CGGTGTGACTCGTGCAGCCA	[35]
Osteocalcin rat	F: ACAGACAAGTCCCACACAGCAACT R: CCTGCTTGGACATGAAGGCTTTGT	[36]
Osteopontin rat	F: CCGATGAATCTGATGAGTCCTT R: TCCAGCTGACTTGACTCATG	[37]
Osteonectin rat	F: GGAAGCTGCAGAAGAGATGG R: TGCACACCTTTTCAAACTCG	[37]
Alkaline phosphatase rat	F: AACCTGACTGACCCTTCCCTCT R: TCAATCCTGCCTCCTTCCACTA	[38]

## Data Availability

Data sharing is not applicable.
